# *N*-heteroatom substitution effect in 3-aza-cope rearrangements

**DOI:** 10.1186/1752-153X-7-94

**Published:** 2013-05-28

**Authors:** Mário JS Gomes, Luis FV Pinto, Paulo MC Glória, Henry S Rzepa, Sundaresan Prabhakar, Ana M Lobo

**Affiliations:** 1 Chemistry Department, REQUIMTE/CQFB, FCT, Universidade Nova de Lisboa, 2829-516, Caparica, Portugal; 2 Departamento das Ciências Naturais e Exatas, Escola Superior de Tecnologia da Saúde de Lisboa, Instituto Politécnico de Lisboa, Lisboa, Portugal; 3 Chemistry Department, Imperial College London, South Kensington Campus, London, UK

## Abstract

**Background:**

The nature of the heteroatom substitution in the nitrogen of a 3-aza-Cope system is explored.

**Results:**

While N-propargyl isoxazolin-5-ones suffer 3-aza-Cope rearrangements at 60°C, the corresponding N-propargyl pyrazol-5-ones need a higher temperature of 180°C for the equivalent reaction. When the propargyl group is substituted by an allyl group, the temperature of the rearrangement for both type of compounds is less affected by the nature of the heteroatom present. Treatment with a base, such as ethoxide, facilitates the rearrangement, and in the case of isoxazol-5- ones other ring opening reactions take precedence, involving N–O ring cleavage of the 5-membered ring. However when base-catalysed decomposition is prevented by substituents, products arising from a room temperature aza-Cope rearrangement are isolated. A possible mechanistic pathway based on free energies derived from density functional calculations involving cyclic intermediates is proposed.

**Conclusions:**

The nature of the heteroatom substitution in the nitrogen of a 3-aza-Cope system leads to a remarkable difference in the energy of activation of the reaction.

## Background

Cope-type rearrangements constitute a highly efficient means for carbon–carbon bond formation, offering high regio- and stereocontrol which renders them especially useful in synthesis [1]. The Aza-Cope rearrangement in particular has some advantages over its oxo-counterpart, namely the fine tuning of the reaction temperature which can be achieved by attaching different substituents to the nitrogen of the rearranging system [2-5], by adding charges or by encapsulating it [6]. As previously reported by us appropriately substituted *N*-alkyl(silyl)oxy-*N*-allyl enamines undergo smooth 3,3-sigmatropic rearrangements to the corresponding *N*-alkyl(silyl)oxy imino ethers in good to excellent yields [7]. Theoretical studies also indicate that an oxyanionic substituent on the nitrogen atom reduces substantially the activation energies for these rearrangements [8]. We report in this manuscript the results obtained with the *N*-propargyl and *N*-allyl 5-membered heterocycles, pyrazolin-5-ones and isoxazolin-5-ones, either under thermolysis or in the presence of a base.

## Results and discussion

**Scheme 1:**
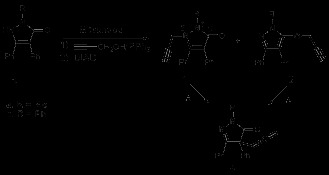
Synthesis and thermal rearrangement of *N*-propargyl pyrazolin-5-ones 1 and their isomers 2. **Synthesis and thermal rearrangement of *N*-propargyl pyrazolin-5-ones 1 and their isomers 2.**

The required compounds **1** for the rearrangement studies were obtained by alkylation of **3** using a Mitsunobu reaction [9] as in Scheme 1. The isolated compounds **1** and **2** were easily separated by chromatography (cf. Experimental). Heating either **1** or **2** gave rise to a 3,3-sigmatropic rearrangement [5,10] and the same final compound **4** as in Scheme 1 (Table 1, entries 1–4).

**Table 1:** Rearrangements of pyrazolin-5-ones 1 and their isomers 2

**#**	**Pyrazolinones 1 & isomers 2**	**Conditions temp./time**	**Products yield**^**a **^**(%)**
1	**1a**: R = Me	180°C / 60 m	**4a**: R = Me (35)
2	**1b**: R = Ph	180°C / 10 m	**4b**: R = Ph (72)
3	**2a**: R = Me	180°C / 10 m	**4a**: R = Me (90)
4	**2b**: R = Ph	180°C / 10 m	**4b**: R = Ph (99)
5	**5a**: R = Me	180°C / 30 m	**6a**: R = Me (30)
6	**7a**: R = Me	180°C / 10 m	**6a**: R = Me (80)

^a^Isolated yields after flash-chromatography.

**Scheme 2:**
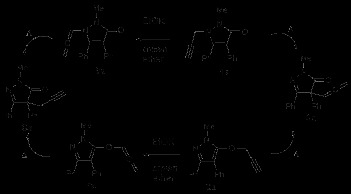
Thermal and base catalyzed rearrangements of pyrazolin-5-ones and their isomers.

In the presence of EtOK and 18-crown-6 ether at r.t., it was found that the propargyl group of **1a** isomerized to the corresponding allene **5a** as in Scheme 2, which could in turn suffer upon heating a 3,3-sigmatropic rearrangement leading to **6a**. This last compound could also be reached from **2a** by treatment with base to give **7a**, followed by thermolysis. Thus coupling of these two reactions opened the way to 3 isomeric compounds of **1a**. It is worth noticing that, while the *N*-allenyl **5a** (Table 1, entry 5) reacts in a similar way to the *N*-propargyl **1a** (entry 1) in the 3-aza-Cope rearrangement, the corresponding *O*-allenyl **7a** (entry 6) and the *O*-propargyl **2a** (entry 3) rearrange faster and give better yields. Similar results are found for the pair **1a**/**2a** and **1b**/**2b**. The reason is probably related to the more facile nature of the 3-oxa-Cope (Claisen) *versus* the 3-aza-Cope rearrangements [3].

**Scheme 3:**
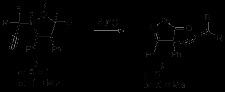
3-Aza-Cope rearrangement of *N*-propargyl isoxazolin-5-ones 8a,b. **3-Aza-Cope rearrangement of *N*-propargyl isoxazolin-5-ones 8a,b.**

**Scheme 4:**
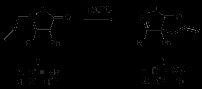
**3-Aza-Cope rearrangement of *N*-allyl isoxazolin-5-ones 8c,d.**

Next, the *N*-propargyl isoxazolinones **8a,b** and the *N*-allyl **8c,d**[11] were synthesised (cf. Experimental) and heated to give the corresponding rearranged compounds, the allenes **9a,b**, (Scheme 3, Table 2, entries 1,2) and the allyl **9c,d** (Scheme 4, Table 2, entries 3,4). It was found that the first ones required a much lower temperature for the rearrangement.

**Table 2:** Thermolysis of isoxazolin-5-ones 8 in the presence and absence of potassium ethoxide

**#**	**Compounds 8**	**Conditions**	**Products Yield**^**a **^**(%)**	
		**Temp./Time**	**9**	**Others**
1	**8a**: R = H	60°C / 30 m	**9a** (75)	-
2	**8b**: R = Me	60°C / 10 m	**9b** (85)	-
3	**8c**: R’ = Me	180°C / 7 h	**9c **(95)^b^	-
4	**8d**: R’ = Ph	180°C / 5 h	**9d** (97)	
5	**8e**	180°C / 5 h	-	**12** (80)
6	**8e**	EtOK^c,d^ / RT / 5 h	-	**13** (80)
7	**8d**: R’ = Ph	EtOK^c,d^ / RT / 10 m	-	**14** (25) **15** (45)
8	**8b**: R = Me	EtOK^c,e^ / RT / 5 d	**9b** (55)	-
		EtOK^c,d^ / RT / 10 m	**9b** (30)	-

^a^Isolated yields after flash-chromatography. ^b^See ref. 11. ^c^In the presence of 18-crown-6 ether. ^d^In the presence of EtOK (1.5 eq). ^e^In the presence of EtOK (0.1 eq).

**Scheme 5:**
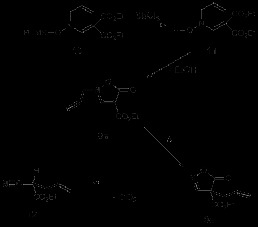
**Synthesis and thermal reaction of *N*-allyl isoxazolin-5-one 8e.**

**Scheme 6:**
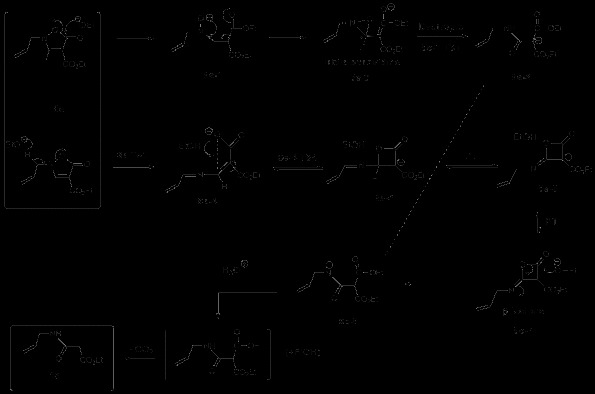
**Base catalyzed reaction of *N*-allyl isoxazolinone 8e.**

The synthesis of **8e** is depicted in Scheme 5 as well as its product after heating (Table 2, entry 5). Desilylation [12] of compound **10** yielded the corresponding ene-hydroxylamine **11**, which was found to be unstable and to lactonise spontaneously to the 2-allyl-isoxazolone **8e** (Scheme 5). The latter on thermolysis (180°C) furnished 2-cyano-pent-4-enoic acid ethyl ester **12**, as shown. In this case the thermodynamic gain resulting from the loss of carbon dioxide from the rearranged product **9e** is also accompanied by the formation of the cyano derivative **12**[13].When treated with potassium ethoxide, **8e** led to the formation of *N*-allyl-malonamic acid ethyl ester **13** instead of the rearranged product (Scheme 6).

Again loss of carbon dioxide, as found earlier by Woodman [14], occurred with a rearrangement possibly involving cyclic intermediates to provide the amide group of **13**[15].The energetics of possible mechanistic pathways for this process were explored using a density functional approach (ωB97XD functional, 6-311G(d,p) basis set and SCRF(CPCM) continuum solvation method for ethanol as a model polar solvent). The reaction is sufficiently complex that a significant number of possible mechanistic pathways can be envisaged using the classical “arrow pushing” approach. Here we adopt the approach of incrementally locating pathways to the product and optimizing them for the lowest overall activation free energy. Such an approach of course does not guarantee finding the global energetic minimum in mechanistic pathways. Instead the objective is to find a thermally reasonable pathway that might approximately correspond to the observed rate of the reaction, and along the way eliminating pathways that have unreasonably high activation free energies.

The mechanistic exploration is set out in Scheme 6. The first route explored involved **8e** and ethoxide anion acting as a nucleophile. Addition of the ethoxide gives **8e-1** as the first intermediate, followed by ring closure to **8e-2**[16]. The most direct route (Occams’s razor) to **13** is by a dyotropic rearrangement of **8e-2** to **8e-3**, followed by protonation and decarboxylation. The energetic high point of this pathway is the transition state for the dyotropic rearrangement of the intermediate oxaziridine (**8e-TS1**) which manifests as unreasonably high (Additional file 1: Table S1, entry 4) ^a^.

The next route explored was with ethoxide acting as a base, abstracting the allylic proton to give the intermediate **8e-4**. A ring closure to **8e-5**, followed by 1,3 intramolecular proton transfer to give **8e-6**, a second proton transfer to reform alkoxide anion and final ring opening to **8e-7** gives **8e-8**, which is merely a tautomer of **8e-3** and can decarboxylate as before to give **13**. The high point of this pathway is **8e-TS2**, which shows as an entirely reasonable barrier for the reaction (Additional file 1: Table S1, entry 5). Whilst this does not constitute a formal mechanistic proof of the reaction, it does imply a reasonable one, and further that any alternative mechanism must have an overall lower energy barrier than this one.

Whereas it is traditional, indeed conventional, for the mechanism of a synthetic pathway to be speculated upon using mechanistic reaction arrows, we suggest here that increasingly such speculations must be supported by a computational exploration of the potential free energy surface. In this case, this has been done with a procedure which, including as it does dispersion corrections for all species, a triple-zeta quality basis with polarization functions, and a correction for continuum solvation, is suggested to be the minimum in quality appropriate for such exploration.

**Scheme 7:**
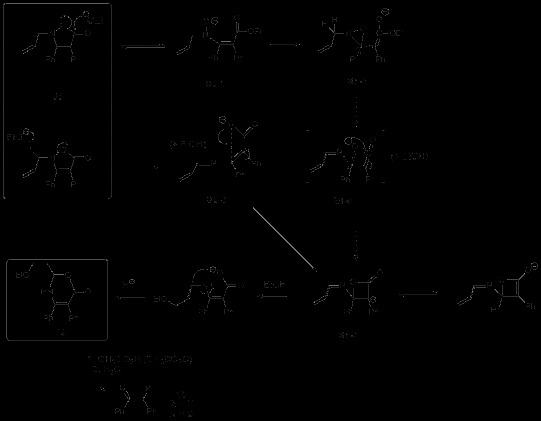
**Base catalyzed reaction of *N*-allyl-3,4-diphenyl-isoxalin-5-one 8d.**

Exposure of 2-allyl-3,4-diphenyl-isoxazolin-5-one (**8d**) to similar conditions gave rise after 10 minutes to **14** in 25% yield (along with desoxybenzoin in 45%, Scheme 7) with no 3-aza-Cope rearrangement being observed. Compound **14** was found to generate desoxybenzoin (**15 R = H**) by quenching under aqueous acidic conditions. Quenching the reaction with deuterated acetic acid (CH_3_COOD) resulted in the isolation of monodeuterated benzoin (**15 R = D**). A possible mechanism is shown and follows along equivalent intermediates, **8d-3** or the oxaziridine derived **8d-4**[17], until reaching **8d-5**. A phenyl substituent on carbon 3 in the β-lactone ring of **8d-5** now leads instead to **14**.

**Scheme 8:**
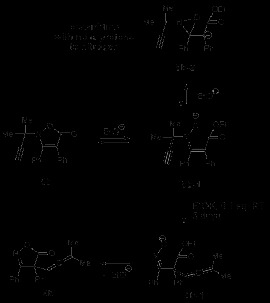
Base catalysed rearrangement of isoxazolin-5-one 8b.

Substrate **8b**, where formation of the carbanion adjacent to the ring nitrogen of the oxazolidinone is blocked by substitution, was next treated with potassium ethoxide (0.1 eq), in the presence of 18-crown-6 ether, at room temperature (5 days), and gave rise to **9b** in 55% yield (cf. Scheme 8). Here the opening of the five membered ring leading to **8b-1** might lower the energy of the reaction either by allowing an easier access to the conformation leading to the transition state or by anionic charge acceleration of the rearrangement. The importance of EtOK was noticed further when, after 5 days at r. t., in its absence, the rearrangement of **8b** was observed to occur, but in a much lower yield (<10%). The same compound treated with LDA or *tert*-BuLi, in the presence of 12-crown-6 ether and in the same conditions of concentration and temperature, showed no reaction. Thus the anion derived from the possible base removal of the terminal triple bond proton does not appear to accelerate the rearrangement, but the oxyanion formed upon the isoxazolidinone ring opening did have a moderately positive effect enabling the rearrangement to occur at room temperature.

## Conclusions

In conclusion: 1) substitution of the nitrogen-1 of pyrazolin-5-ones by oxygen lowers the temperature of the 3-aza-Cope rearrangement of isoxazolin-5-ones vis-à-vis the corresponding pyrazolin-5-ones when the rearranging element includes a propargyl group attached to the heterocyclic nitrogen-2; 2) when the propargyl group is replaced by an allyl group the rearranging temperature is higher and similar in both compounds; 3) treatment with potassium ethoxide as base in the presence of 12-crown-6 ether at room temperature, while leaving the pyrazolin-5-one heterocycle untouched, leads to ring opening of isoxazolin-5-ones, followed by further reactions. The putative oxaziridine or other cyclic intermediate then suffer base catalysed ring opening reaction whenever there are protons available in the carbon α to the ring nitrogen, leading to a 6-membered aza,oxa-ring system. When such position is blocked by substituents the 3-aza-Cope rearrangement occurs at room temperature.

### Experimental

#### *General*

Melting points were determined on a Reichert Thermovar apparatus and are uncorrected. Ordinary mass spectra were recorded on a Fisons TRIO 2000 or AEI MS-9 spectrometer. High-resolution MS spectra (HRMS) were obtained on a FT- ICR/ MS Finnigan FT/MS 2001-DT spectrometer at 70 eV by electron impact or on a Finnigan MAT 900 ST spectrometer by ESI. Infrared (IR) spectra were recorded on a Perkin–Elmer 1000X FT-IR spectrometer. Proton and ^13^C NMR spectra were recorded in CDCl_3_ on a Bruker ARX 400 spectrometer (400 MHz for ^1^H, 100.63 MHz for ^13^C). Chemical shifts are reported relative to tetramethylsilane as the internal reference (δ_H_ 0.00) for ^1^H NMR spectra and to CDCl_3_ (δ_C_ 77.00) for ^13^C NMR spectra. IR spectra were run on an FT Perkin–Elmer 1000 instrument, with absorption frequencies expressed in reciprocal centimeters. Thin-layer chromatography was performed on Merck silica gel 60 F_254_ 0.2 mm thick plates, visualized under UV light or by exposing to iodine vapour. For preparative separations the plates were 0.5–1 mm thick. For flash chromatography silica Merck Kieselgel 60, 70–230 mesh was used. Usual work-up implies drying the water- or brine-washed organic extracts over anhydrous sodium sulfate or magnesium sulfate, followed by filtration and solvent removal under reduced pressure. Anhydrous solvents were dried and freshly distilled by standard methods [18].

### Synthesis of starting materials

#### *Ethyl 3-oxo-2-phenyl-butanoate*

To a solution of ethyl acetoacetate (1.5 g, 11.5 mmol) in CH_2_Cl_2_ (45 mL), was added triphenylbismuth carbonate [19] (6.33 g, 12.6 mmol). After being stirred under argon for 24 h at 40°C, the mixture was filtered and concentrated at reduced pressure. The crude was purified by CC (silica; Et_2_O / *n*-hexane 1:3) to give the title compound as a yellow oil [20] (1.44 g, 61%): IR (NaCl) ν_max_ (cm^-1^): 3063, 2983, 1745 (C = O, ester), 1721 (C = O, ketone). ^1^H NMR (400 MHz CDCl_3_) *keto-enol* equilibrium, *enol* (55%): *δ* 1.18 (3H, t, *J* = 7.2 Hz, OCH_2_C*H*_3_), 1.85 (3H, s, CH_3_CO), 4.23 (2H, m, OC*H*_2_CH_3_), 7.14 – 7.38 (5H, m, Ar–H), 13.13 (1H, s, OH); *ketone* (45%); 1.27 (3H, t, *J* = 7.2 Hz, OCH_2_C*H*_3_), 2.18 (3H, s, CH_3_CO), 4.23 (2H, m, OC*H*_2_CH_3_), 4.69 (1H, s, PhCH), 7.14 – 7.38 (5 H, m, Ar–H).

#### *Ethyl 3-oxo-2,3-diphenyl-propanoate*

To a solution of ethyl 3-oxo-3-phenyl-propionate (1.48 g, 7.7 mmol) in CH_2_Cl_2_ (25 mL), was added triphenylbismuth carbonate (4.22 g, 8.5 mmol). After being stirred under argon for 24 h at 40°C, the mixture was filtered and concentrated at reduced pressure. The crude was purified by CC (silica; Et_2_O / *n*-hexane 1:3) to give a viscous oil. Recrystallization from ethanol yielded the title compound (1.34 g, 65%): mp 88–89°C (ethanol) (lit.: [21] mp 89–90°C). IR (KBr) *ν*_max_ (cm^-1^): 3063, 2985, 1741 (C = O, ester), 1674 (C = O, ketone).^1^H NMR (400 MHz CDCl_3_) *keto-enol* equilibrium, *enol* (12%): *δ* 1.25 (3H, t, *J* = 7.2 Hz, OCH_2_C*H*_3_), 4.23 (2H, m, OC*H*_2_CH_3_), 7.11 – 7.99 (10 H, m, Ar–H), 13.66 (1H, s, OH), and *ketone* (88%): *δ* 1.25 (3H, t, *J* = 7.2 Hz, OCH_2_C*H*_3_), 4.23 (2H, m, OC*H*_2_CH_3_), 5.62 (1H, s, PhCH), 7.11 – 7.99 (10 H, m, Ar–H).

#### *Methyl-4-phenylisoxazolin-5-one*

To a solution of ethyl 3-oxo-2-phenyl-butanoate (50 mg, 0.24 mmol) in EtOH (0.5 mL) and H_2_O (0.1 mL), was added hydroxylamine hydrochloride (32 mg, 0.46 mmol) and sodium acetate (6.4 mg, 0.078 mmol). The solution was refluxed with continuous stirring under argon for 3 h, 37% HCl (32 μL) was added and the mixture refluxed for further 30 min. The solvent was removed under reduced pressure and the crude diluted with distilled water (2 mL) and extracted with Et_2_O (3 × 5 mL). The organic extract was dried, filtered and concentrated under reduced pressure. Purification by CC (silica; AcOEt) yielded the title compound (32 mg, 66%): mp 138–140°C (ethyl acetate) (lit.: [22] mp 139–140°C). IR (KBr) *ν*_max_ (cm^-1^): 3322 (NH), 1669 (C = O). ^1^H NMR (400 MHz CDCl_3_) two distinct forms, isoxazol-5(2H)-one (**A**) and isoxazole-5(4H)-one (**B**) (**A**:**B** = 90:10) **A**, *δ* 2.35 (3H, s, CH_3_), 6.24 (1H, sl, NH), 7.14 – 7.56 (5H, m, Ar–H); **B**, *δ* 2.04 (3H, s, CH_3_), 4.40 (1H, s, CH), 7.14 – 7.56 (5H, m, Ar–H).

#### *Diphenylisoxazolin-5-one*

To a solution of ethyl 3-oxo-2,3-diphenyl-propanoate (65 mg, 0.27 mmol) in EtOH (0.5 mL) and H_2_O (0.1 mL), was added hydroxylamine hydrochloride (32 mg, 0.46 mmol) and sodium acetate (6.4 mg, 0.078 mmol). The solution was refluxed with continuous stirring under argon for 3 h and processed as in the previous reaction to yield the title compound (40 mg, 61%) as a white powder: mp: 159°C (dec.) (benzene) (lit.: [23] mp 159°C). IR (KBr) *ν*_max_ (cm^-1^): 3297 (NH), 1680 (C = O). ^1^H NMR (400 MHz CDCl_3_) *δ* 5.86 (1H, sl, NH), 7.01 – 7.79 (10H, m, Ar–H).

### General procedure for the synthesis of pyrazolinones

To a stirred ethanolic solution (≈ 0.5 M) of ethyl 3-oxo-2,3-diphenylpropanoate [21] (1 eq.) in ethanol is added the desired hydrazine (1 eq.) and the reaction heated to reflux under argon. After total consumption of both starting materials (TLC control: silica, CH_2_Cl_2_:MeOH, 9:1), the solvent is evaporated under reduced pressure and the product is purified by hot recrystallization using AcOEt.

#### *Diphenyl-1-methyl-2-pyrazolin-5-one (3a)*

From methyl hydrazine, compound **3a** obtained after 18 h as colourless crystals (50%): mp: 214–216°C (AcOEt). ^1^H NMR (400 MHz CDCl_3_) δ 7.72 – 7.20 (10H, m, Ar–H), 3.70 (3H, s, NCH_3_). ^13^C NMR (100.62 MHz CDCl_3_) δ 36.4 (NCH_3_), 108.4 , 124.9, 126.9, 127.2, 129.0, 131.8, 145.3, 165.8 (C = O). IR (KBr; cm^-1^): 3235 (N-H),1695 (C = O). EIMS (m/z, %): 250 (M^+^, 100), 178 (58), 77 (26). HRMS: calcd for C_16_H_14_N_2_O: 250.11076; found: 250.11061.

#### *Triphenyl-2-pyrazolin-5-one (3b)*

From phenylhydrazine, compound **3b** obtained after 24 h as colourless crystals (71%): mp 198–199°C (AcOEt). ^1^H NMR (400 MHz DMSO-d_6_ / CDCl_3_, 1:2) δ 10.97 (1H, bs, OH), 7.84 – 7.25 (15H, m, Ar–H). IR (KBr, cm^-1^): 3236 (N-H), 1697 (C = O). EIMS (m/z, %): 312 (M^+^, 78), 178 (66), 77 (100). HRMS: calcd for C_21_H_16_N_2_O: 312.12626; found: 312.12661.

#### *Mitsunobu propargylation of pyrazolinones 3*

General procedure: To the desired pyrazolone (**3a** or **3b**) (1.0 eq.) dissolved in dry THF (≈ 0.15 M) under argon, PPh_3_ (1.2 eq.) and the required propargyl alcohol (1.0 eq.) are added. The reaction is cooled to 0°C and DIAD (1.2 eq.) in dry THF (≈ 1.5 M) is added dropwise. The reaction is allowed to reach room temperature and after the reaction is finished (tlc control), the solvent is removed under vacuum. The products are purified by flash chromatography [AcOEt/*n*-hexane, (1:1) → AcOEt]. Following the general procedure, compounds (**1a**) and (**2a**) were obtained from **3a**, and **1b** and **2b** from **3b**.

#### *Diphenyl-1-methyl-2-propargylpyrazolin-5-one (1a)*

(65%), colourless crystals, mp 123–124°C (AcOEt). ^1^H NMR (400 MHz CDCl_3_) *δ* 7.47 – 7.34 (6H, m, Ar–H), 7.24 – 7.15 (4H, m, Ar–H), 4.14 (2H, d, *J =* 2.0 H*z*, NC*H*_2_C ≡ CH), 3.49 (3H, s, NCH_3_), 2.21 (1H, t, *J* = 2.0 Hz, NCH_2_C ≡ C*H*). ^13^C NMR (100.62 MHz CDCl_3_) *δ* 165.5 (C = O), 152.7 [N*C*(Ph)], 130.4 , 130.0, 129.4, 129.0, 129.0, 128.7, 127.9, 126.7, 115.2, 75.4, 74.5, 39.0, 29.2. I.R. (NaCl; cm^-1^): 3279, 2109, 1651 (C = O). EIMS (m/z, %): 288 (M^+^, 46), 277 (100), 249 (C_16_H_13_N_2_O^+^, 25), 199 (32); 77 (45). HRMS: calcd for C_19_H_16_N_2_O: 288.126263; found: 288.126523.

#### *3,4-Diphenyl-1-methyl-5-propargyloxy-pyrazole (2a)*

(35%), colourless oil. ^1^H NMR (400 MHz CDCl_3_) *δ* 7.46 – 7.43 (2H, m, Ar–H), 7.34 – 7.22 (8H, m, Ar–H), 4.40 (2H, d, *J =* 2.4 Hz, OC*H*_2_C ≡ CH), 3.86 (3H, s, NCH_3_), 2.49 (1H, t, *J =* 2.4 Hz, OCH_2_C ≡ C*H*).^13^C NMR (100.62 MHz CDCl_3_) *δ* 149.8 [N = *C*(Ph)], 147.5 [N = C(Ph)C(Ph) = *C*], 133.7 (Ar), 132.0 (Ar), 129.6 (Ar), 128.5 (Ar), 128.1 (Ar), 127.8 (Ar), 127.4 (Ar), 126.8 (Ar), 105.6 [N = C(Ph)*C*(Ph) = C], 77.7 (OCH_2_*C* ≡ CH), 76.7 (OCH_2_C ≡ *C*H), 61.0 (O*C*H_2_C ≡ CH), 34.6 (NCH_3_). I.R. (NaCl, cm^-1^): 3289, 2123. EIMS (m/z, %): 288 (M^+^, 40), 249 (C_16_H_13_N_2_O^+^, 100), 221 (18), 178 (5), 115 (25), 91 (20). HRMS: calcd for C_19_H_16_N_2_O: 288.126263; found: 288.126219.

#### *2-Propargyl-1,3,4-triphenyl-2-pyrazolin-5-one (1b)*

(35%), yellowish oil. ^1^H NMR (400 MHz CDCl_3_) *δ* 7.65 (2H, d, *J =* 7.6 Hz, Ar–H), 7.52 – 7.41 (8H, m, Ar–H), 7.33 – 7.19 (5H, m, Ar–H), 4.03 (2H, d, *J =* 2.2 Hz, NC*H*_2_C ≡ CH), 2.16 (1H, t, *J* = 2.2 Hz, NCH_2_C ≡ C*H*). ^13^C NMR (100.62 MHz CDCl_3_) *δ* 164.6 (C = O) , 154.9 [N*C*(Ph) = C(Ph)] , 134.8 (Ar) , 130.2 (Ar), 130.1 (Ar), 129.5 (Ar), 129.2 (Ar), 129.0 (Ar), 129.0 (Ar), 128.0 (Ar), 127.0 (Ar), 126.6 (Ar), 123.5 (Ar), 116.6 [NC(Ph) = *C*(Ph)], 74.6 (NCH_2_*C* ≡ CH), 74.1 (NCH_2_C ≡ *C*H), 40.6 (N*C*H_2_C ≡ CH). IR (film, cm^-1^): 3298, 2116, 1667 (C = O).

#### *5-Propargyloxy-1,3,4-triphenyl-2-pyrazole (2b)*

(35%), yellowish oil. ^1^H NMR (400 MHz CDCl_3_) *δ* 7.91 (2H, d, *J =* 7.8 Hz, Ar–H), 7.58 – 7.30 (13H, m, Ar–H), 4.40 (2H, d, *J =* 2.3 Hz, OC*H*_2_C ≡ CH), 2.36 (1H, t, *J = 2.3 Hz*, OCH_2_C ≡ C*H*). ^13^C NMR (100.62 MHz CDCl_3_) *δ* 149.5 [N = *C*(Ph)C(Ph) = C], 149.0 [N = C(Ph)C(Ph) = *C*], 138.5 (Ar), 133.3 (Ar), 131.6 (Ar), 129.9 (Ar), 128.9 (Ar), 128.5 (Ar), 128.1 (Ar), 128.0 (Ar), 127.7 (Ar), 127.1 (Ar), 126.8 (Ar), 122.8 (Ar), 107.9 [N = C(Ph)*C*(Ph) = C], 77.1 (OCH_2_*C* ≡ CH), 76.8 (OCH_2_C ≡ *C*H), 61.0 (O*C*H_2_C ≡ CH). IR (film, cm^-1^): 3291, 2125.

### General procedure for the thermal rearrangements of pyrazolinones and isomers

In a round bottom flask, the *N-*substituted pyrazolinone or its *O-*substituted isomer dissolved in *o*-dichlorobenzene (ca. 0.04 – 0.05 M), are heated at 180°C until total consumption of the starting material (TLC control). After solvent evaporation the products are purified by flash chromatography [Et_2_O/*n*-hexane (1:4)].

#### *Pyrazolinone 4a from 1a*

Following the general procedure, heating **1a** (30 mg) for 60 min afforded 3,4-diphenyl-1-methyl-4-(prop-1′,2′-dienyl) pyrazolin-5-one (**4a**) (10.5 mg, 35%) as a colourless crystals: mp 146–147°C (Et_2_O). ^1^H NMR (400 MHz CDCl_3_) *δ* 7.56 (2H, d, *J* = 7.0 Hz, Ar–H), 7.37 – 7.26 (8H, m, Ar–H), 5.79 (1H, t, *J* = 6.6 Hz, (C*H* = C = CH_2_), 4.90 (1H, dd, *J* = 11.6, 6.6 Hz, CH = C = C*H*_2_), 4.74 (1H, dd, *J* = 11.6, 6.6 Hz, CH = C = C*H*_2_), 3.45 (3H, s, NCH_3_).^13^C NMR (100.62 MHz CDCl_3_) *δ* 208.9, 174.5, 158.8, 136.1, 129.9, 129.2, 128.4, 128.2, 127.0, 89.1, 79.1, 60.1, 31.8. IR (film, cm^-1^ ) : 3061, 2921, 1956 (C = C = C), 1714 (C = O), 1496.EIMS (m/z, %): 288 (M^+^, 60), 230 (40), 178 (20), 128 (65), 77 (100). HRMS: calcd for C_19_H_16_N_2_O: 288.126263; found: 288.126455.

#### *Pyrazolinone 4b from 1b*

Following the general procedure, heating **1b** (14 mg) for 10 min afforded 4-(prop-1′,2′-dienyl)-1,3,4-triphenylpyrazolin-5-one (**4b**) (10.1 mg, 72%) as a colourless oil. ^1^H NMR (400 MHz CDCl_3_) *δ* 8.07 (2H, d, *J* = 7.8 Hz, Ar–H), 7.70 (2H, d, *J* = 7.8 Hz, Ar–H), 7.47 – 7.23 (11H, m, Ar–H), 5.90 (1H, t, *J* = 6.7 Hz, C*H* = C = CH_2_), 4.94 (1H, dd, *J* = 11.7, 6.7 Hz, CH = C = C*H*_2_), 4.77 (1H, dd, *J =* 11.7, 6.7 Hz, CH = C = C*H*_2_). ^13^C NMR (100.62 MHz CDCl_3_) *δ* 209.0 (CH = *C* = CH_2_), 172.7 (C = O), 159.1 (N = C), 138.2, 136.0, 130.2, 130.1, 129.3, 128.9, 128.4, 127.4, 127.0, 125.3, 119.1, 89.1 (*C*H = C = CH_2_), 79.5 (CH = C = *C*H_2_), 61.6 (*C*–CH = C = CH_2_). IR (film, cm^-1^): 3063, 2921, 1954 (C = C = C), 1721 (C = O), 1596, 1494. EIMS (m/z, %): 350 (M^+^, 25), 230 (12), 178 (10), 128 (21), 91 (32), 77 (100). HRMS: calcd for C_24_H_18_N_2_O: 350.141913; found: 350.142194.

#### *Pyrazolinone 4a from isomer 2a*

Following the general procedure, heating **2a** (20 mg) for 10 min afforded 3,4-diphenyl-1-methyl-4-(prop-1′,2′-dienyl)pyrazolin-5-one (**4a**) (18 mg, 90%) as a white solid with data identical with *4.4.1*.

#### *Pyrazolinone 4b from isomer 2b*

Following the general procedure, heating **2b** (24 mg) for 10 min afforded 4-(prop-1′,2′-dienyl)-1,3,4-triphenylpyrazolin-5-one (**4b**) (24 mg, 99%) as a white solid with data identical with *4.4.2*.

#### *Pyrazolinone 6a from 5a*

Following the general procedure, heating **5a** (10 mg) for 30 min afforded the pyrazolinone **6a** (3 mg, 30%) as a glassy solid. ^1^H NMR (400 MHz CDCl_3_) *δ* 7.52 (2H, d, *J* = 7.3 Hz, Ar–H), 7.38 – 7.22 (8H, m, Ar–H), 3.47 (3H, s, NCH_3_), 3.43 (1H, dd, *J* = 16.0 Hz, 2.5 Hz, C*H*_2_C ≡ CH), 3.05 (1H, dd, *J* = 16.0, 2.5 Hz, C*H*_2_C ≡ CH), 1.91 (1H, t, *J =* 2.5 Hz, CH_2_C ≡ C*H*). IR (film, cm^-1^): 3291 (C ≡ C-H), 2923, 1956 (C ≡ C), 1713 (C = O).

#### *Pyrazolinone 6a from isomer 7a*

Following the general procedure, heating **7a** (10 mg) for 10 min afforded **6a** (8 mg, 80%) with data identical with the previous reaction.

### Reactions of pyrazolinones with base

In a round bottom flask, the propargylated pyrazolinone **1a** or its isomer **2a** is dissolved in dry THF (ca. 0.07 M) (1 eq.) under argon and 18-crown-6 ether (1 eq.) is added. After total dissolution of the reactants, EtOK (0.1 eq.) is added under anhydrous conditions. The reaction is left to react at room temperature. After consumption of the starting material (TLC control) the reaction is stopped by addition of a saturated solution of NH_4_Cl and extraction with Et_2_O (2x). The product is purified by flash chromatography [AcOEt / *n*-hexane (1:6)].

#### *Pyrazolinone 6a from isomer 7a*

Following the general procedure, 3,4-diphenyl-1-methyl-2-(prop-1′,2′-dienyl)pyrazolin-5-one (**5a**) was obtained from **1a** after 16 h as a colourless oil (65%). ^1^H NMR (400 MHz CDCl_3_) *δ* 7.69 – 7.64 (4H, m, Ar–H), 7.56 – 7.52 (2H, m, Ar–H), 7.47 – 7.43 (4H, m, Ar–H), 6.30 (1H, t, *J* = 6.2 Hz, N–C*H* = C = CH_2_), 5.32 (2H, d, *J* = 6.2 Hz, NCH = C = C*H*_2_), 3.49 (3H, s, NCH_3_). IR (film, cm^-1^): 3055, 2925, 1962 (C = C = C), 1659 (C = O), 1483.

#### *Isomer 7a from 2a*

Following the general procedure, 1-methyl-3,4-diphenyl-5-(prop-1′,2′-dienyloxy)pyrazole (**7a**) was obtained from **2a** after 16 h as a colourless oil (74%). ^1^H NMR (400 MHz CDCl_3_) *δ* 7.47 – 7.43 (2H, m, Ar–H), 7.34 – 7.24 (8H, m, Ar–H), 6.79 (1H, t, *J* = 5.9 Hz, OC*H* = C = CH_2_), 5.36 (2H, d, *J* = 5.9 Hz, OCH = C = C*H*_2_), 3.81 (3H, s, NCH_3_). ^13^C NMR (100.62 MHz CDCl_3_) *δ* 200.0 (OCH = *C* = CH_2_), 147.7, 147.5, 133.5 (Ar), 131.6, 129.6, 129.2, 128.3, 128.2, 127.9, 127.6, 126.7, 121.7 (O*C*H = C = CH_2_), 93.0 (OCH = C = *C*H_2_), 34.7 (NCH_3_). IR (film, cm^-1^): 3059, 2940, 1972 (C = C = C), 1605, 1563.

### Synthesis of isoxazolinones

#### *Diphenyl-2-(prop-2′-yn-1′-yl)isoxazolin-5-one (8a)*

To a solution of 3,4-diphenylisoxazolin-5-one (Section 4.2.4) (100 mg, 0.422 mmol), triphenylphosphine (121 mg, 0.46 mmol) and propargylic alcohol (21.5 mg, 0.384 mmol) in dry THF (1.5 mL) were added, dropwise, DIAD (93 mg, 0.460 mmol). After the addition was completed, the solvent was removed under reduced pressure and the crude purified by CC (silica; Et_2_O / *n*-hexane 1:2) to yield the title compound **8a** (22 mg, 21 %): mp 81–82°C (Et_2_O / *n*-hexane). IR (KBr, cm^-1^): 3291 (HC≡C), 2127 (C≡C), 1746 (C = O). ^1^H NMR (400 MHz CDCl_3_) *δ* 2.64 (1H, s, HC≡C), 5.06 (2H, d, *J* = 2.1 Hz, NCH_2_), 7.26 – 7.56 (10H, m, Ar–H). EIMS (m/z, %): 275 (M^+^, 23), 178 (34), 105 (100), 89 (60), 77 (50). HRMS calcd for C_18_H_13_NO_2_: 275.09463; found: 275.09405.

#### *Diphenyl-2-(2′-methylbut-3′-yn-2′-yl)isoxazolin-5-one (8b)*

Following Nicholas protocol [24], to a stirred solution of 2-methylbut-3-yn-2-ol (25.2 mg, 0.3 mmol) in petroleum ether (2 mL), under nitrogen at RT, was added Co_2_(CO)_8_ (102.6 mg, 0.3 mmol.) and MS 4 Å, followed by boron trifluoride diethyl etherate (42.5 mg, 0.3 mmol). After 10 min 3,4-diphenylisoxazolin-5-one (Section 4.2.4) (23.7 mg, 0.1 mmol) was added and the reaction monitored by TLC (silica; CH_2_Cl_2_ / AcOEt 4:1) until consumption of the starting isoxazolone. After 48 h water (5 mL) was added to the reaction mixture which was extracted with Et_2_O (2x5 mL) to yield the cobalt complex as a red oil, which was purified by PTLC (silica; *n*-hexane / AcOEt 4:1). To a solution of this red complex in acetone (2 mL) was added Et_3_N (0.1 mmol) and (NH_4_)_2_Ce(NO_3_)_6_ (55 mg, 0.1 mmol) until formation of the required isoxazolinone. Water was added (10 mL), followed by extraction of the mixture with Et_2_O (3x5 mL) and purification by PTLC (silica; Et_2_O / *n*-hexane 1:1) afforded the *N*-substituted isoxazolinone **8b** (17.6 mg, 58%): mp 113–115°C (Et_2_O / *n*-hexane). IR (KBr, cm^-1^): 3291 (HC≡C), 2113 (C≡C), 1746 (s, C = O). ^1^H NMR (400 MHz CDCl_3_) *δ* 1.48 (6H, s, CH_3_), 2.31 (1H, s, HC≡C), 7.19 – 7.50 (10H, m, Ar–H). ^13^C-NMR (100.62 MHz CDCl_3_) *δ* 28.5 [NC(*C*H_3_)_2_], 61.3 [N*C*(CH_3_)_2_], 73.7 (H*C*≡C), 82.4 (HC≡*C*), 127.4, 128.2, 128.3, 128.7, 129.5, 130.6, 170.2. EIMS (m/z, %): 303 (M^+^, 17), 237 (C_15_H_11_NO_2_^+^, 100), 178 (35), 67 (40). HRMS: calcd for C_20_H_17_NO_2_: 303.12593; found: 303.12641.

#### *Allyl-3-methyl-4-phenylisoxazolin-5-one (8c)*

To 3-methyl-4-phenylisoxazolin-5-one (Section 4.2.3) (0.3 g, 1.71 mmol) was added a solution of Na (39 mg) in EtOH (1.7 mL). To this solution was added allyl bromide (0.207 mg, 1.71 mmol). The reaction mixture was magnetically stirred, under argon, while being heated under reflux (1.8 h). On completion of the reaction (TLC control: silica; 1) Et_2_O / *n*-hexane 1:1, 2) AcOEt), the solvent was removed under reduced pressure. Purification by CC (silica; Et_2_O / *n-*hexane 1:1) afforded the title compound **8c** (171,6 mg, 66%) as a white solid: mp 50–51°C **(** Et_2_O / *n*-hexane). IR (KBr, cm^-1^): To 3-methyl-4-phenylisoxazolin-5-one (Section 4.2.3) (0.3 g, 1.71 mmol) was added a solution of Na (39 mg) in EtOH (1.7 mL). To this solution was added allyl bromide (0.207 mg, 1.71 mmol). The reaction mixture was magnetically stirred, under argon, while being heated under reflux (1.8 h). On completion of the reaction (TLC control: silica; 1) Et_2_O / *n*-hexane 1:1, 2) AcOEt), the solvent was removed under reduced pressure. Purification by CC (silica; Et_2_O / *n-*hexane 1:1) afforded the title compound **8c** (171,6 mg, 66%) as a white solid: mp 50–51°C ( Et_2_O / *n*-hexane). IR (KBr, cm^-1^): 1711 (C = O), 1604 (C = C).^1^H NMR (400 MHz CDCl_3_) *δ* 2.29 (3H, s, CH_3_), 4.27 (2H, d, J 6 Hz, NCH_2_), 5.30 (2H, m, C*H*_2_ = CH), 5.80 (1H, m, CH_2_ = C*H*), 7.27 (1H, t, *J* = 7.4 Hz, *p*-Ar–H), 7.38 (2H, t, *J* = 7.5 Hz, *m*-Ar–H), 7.46 (2H, d, *J* = 7.6 Hz, *o*-Ar–H). ^13^C-NMR (100.62 MHz CDCl_3_) *δ* 11.8 (CH_3_), 53.1 (N–CH_2_), 103.8 [N–C(Me) = *C*(Ph)], 120.5 (*C*H_2_ = CH), 127.2 (Ar), 128.2 (Ar), 128.5 (Ar), 129.4 (Ar), 129.6 (CH_2_ = *C*H), 159.3 [N–*C*(Me) = C(Ph)], 169.5 (C = O). EIMS (m/z, %): 215 (M^+^, 100), 130 (21), 116 (38), 115 (46), 89 (13), 77 (13). HRMS: calcd for C_13_H_13_NO_2_: 215.09463; found: 215.09397.

#### *Allyl-3,4-diphenylisoxazolin-5-one (8d)*

3,4-Diphenylisoxazolin-5-one (Section 4.2.4) (0.406 g, 1.71 mmol), using the previous protocol, afforded the title compound (**8d**) (256 mg, 85%) as a white solid: mp 105–106°C (Et_2_O / *n*-hexane). IR (KBr, cm^-1^): 1720 (C = O), 1615 (C = C). ^1^H NMR (400 MHz CDCl_3_) *δ* 4.06 (2H, d, *J* 6.0 Hz, NCH_2_), 5.16 (1H, *d*, *J* = 17.2 Hz, C*H*_2_ = CH), 5.25 (1H, d, *J* = 10.8 Hz, C*H*_2_ = CH), 5.78 (1H, m, CH_2_ = C*H*), 7.18 – 7.48 (10 H, m, Ar–H). ^13^C NMR (100.62 MHz CDCl_3_) *δ* 54.9 (N–CH_2_), 105.2 [N–C(Ph) = *C*(Ph)], 121.0 (*C*H_2_ = CH), 127.2 (Ar), 127.7 (Ar), 128.2(Ar), 128.3(Ar), 128.7(Ar), 129.0(Ar), 129.2(Ar), 129.3(Ar), 131.0 (CH_2_ = *C*H), 162.8 [N–*C*(Ph)], 169.9 (C = O). EIMS (m/z, %): 277 (M^+^, 100), 236 (12), 192 (27), 178 (76), 133 (13), 117 (51), 89 (46), 77 (22). HRMS: calcd for C_18_H_15_NO_2_: 277.11028; found: 277.11037.

#### *Synthesis of isoxazolidinone 8e*

##### Diethyl 2-[(allyl-(tert-butyldimethylsilyloxy)amino) methylene]malonate (10)

To a stirred solution of *N*-*tert*-butyldimethylsilyloxyprop-2-en-1-amine (0.5 g, 2.67 mmol) in CCl_4_ / CHCl_3_ (1:3) (20 mL) under argon was added diethyl 2-(ethoxymethylene)malonate [7] (0.58 g, 2.67 mmol, 1 eq.). On completion of the reaction (TLC control: silica, Et_2_O / *n*-hexane 1:1) (30 h), the solvent was removed under reduced pressure by CC (silica; Et_2_O / *n*-hexane 1:1) to afford the title compound **10** (50%) as an oil (50%): IR (film, cm^-1^) 1732 (C = O), 1707 (C = O), 1621 (C = C), 1262 (Si–O-N). ^1^H NMR (400 MHz CDCl_3_) *δ* 0.19 [6H, s, Si(CH_3_)_2_], 0.93 [9H, s, C(CH_3_)_3_], 1.23 (6H, m, OCH_2_C*H*_3_), 4.00 (2H, d, *J* = 6.0 Hz, NCH_2_), 4.12 (4H, m, OC*H*_2_CH_3_), 5.26 (1H, d, *J* = 10.4 Hz, C*H*_2_ = CH), 5.35 (1H, d, *J* = 17.3 Hz, C*H*_2_ = CH), 5.90 (1H, m, CH_2_ = C*H*), 7.88 (1H, s, N–CH = C). ^13^C NMR (100.62 MHz CDCl_3_) *δ* -5.3 [Si(CH_3_)_2_], 14.1 (OCH_2_*C*H_3_), 17.8 [*C*(CH_3_)_3_], 25.8 [C(*C*H_3_)_3_], 59.9 (CH_2_N), 60.3 (O*C*H_2_CH_3_), 60.6 (O*C*H_2_CH_3_), 61.4 (NCH = *C*), 119.6 (*C*H_2_ = CH), 131.1 (CH_2_ = *C*H), 149.5 (N–CH), 166.2 (C = O). EIMS (m/z, %): 357 (M^+^, 66), 312 ([M-C_2_H_5_O]^+^, 86), 300 ([M-C_4_H_9_]^+^, 100), 226 ([M-C_6_H_15_OSi]^+^, 97), 198 (20), 57 (C_4_H_9_^+^, 49). HRMS: calcd for C_17_H_31_NO_5_Si: 357.19715; found: 357.19796.

##### Diethyl 2-[(allyl-(tert-butyldimethylsilyloxy)amino) methylene]malonate (10)

To a magnetically stirred solution of 2-[(allyl-(*tert*-butyldimethylsilyloxy)amino) methylene]malonate [5] (**10**) (50 mg, 0.140 mmol) in CH_3_CN [6 mL, 0.1% H_2_O (m/v)], at RT under nitrogen, was added SbCl_5_ (359 μL, from 0.039 M solution in CH_3_CN, 0.1 eq.). On completion of the reaction (10 m), (TLC control: silica; Et_2_O / *n*-hexane 1:1), the solvent was removed under reduced pressure. Purification by CC (silica; Et_2_O / *n*-hexane 1:1) afforded diethyl 2-[(allyl(hydroxyl)amino) methylene]malonate (**11**) (23 mg, 68%), and ethyl 2-allyl-5-oxo-2,5-dihydroisoxazole-4-carboxylate (**8e**) (3.3 mg, 12%). Compound **11** was unstable and lactonised spontaneously on standing to the 2-allyl-isoxazolinone **8e**.

##### Ethyl 2-allyl-5-oxo-2,5-dihydroisoxazole-4-carboxylate (8e)

Colourless crystals, mp 45–46°C (*n*-hexane). IR (film, cm^-1^): 1784 (C = O, ester), 1698 (C = O), 1578 (C = C). ^1^H NMR (400 MHz CDCl_3_) *δ* 1.30 (3H, t, *J* = 7.1 Hz, OCH_2_C*H*_3_), 4.26 (2H, q, *J* = 7.1 Hz, OC*H*_2_CH_3_), 4.47 (2H, d, *J* = 6.3 Hz, NC*H*_2_), 5.46 (2H, m, C*H*_2_ = CH), 5.89 (1H, m, CH_2_ = C*H*), 8.34 (H, s, N–CH). Elemental analysis: calcd for C_9_H_11_NO_4_ (%): C: 54.80, H: 5.63, N: 7.11. Found (%): C: 54.53, H: 5.66, N: 7.01.

##### *Diethyl 2-[(N-allyl(hydroxyl)amino)methylene] malonate* (11)

Oil, IR (film, cm^-1^): 3600 – 1800 (l, OH), 1685 (C = O), 1629 (C = C). ^1^H NMR (400 MHz CD_3_CN) *δ* 1.26 (3H, t, *J* = 7.1 Hz, OCH_2_C*H*_3_), 1.33 (3H, t, *J* = 7.1 Hz, OCH_2_C*H*_3_), 4.16 (2H, q, *J* = 7.1 Hz, OC*H*_2_CH_3_), 4.25 (4H, m, OC*H*_2_CH_3_ + NC*H*_2_), 5.39 (2H, m, C*H*_2_ = CH), 5.96 (1H, m, CH_2_ = C*H*), 7.86 (1H, s, N–*C*H = C), 13.11 (1H, s, OH, exchange D_2_O). ^13^C NMR (100.62 MHz CD_3_CN) *δ* 14.1 (OCH_2_*C*H_3_), 30.2 (*C*CO_2_Et), 60.0 (O*C*H_2_CH_3_), 61.5 (O*C*H_2_CH_3_), 61.6 (NCH_2_), 120.8 (CH = *C*H_2_), 129.9 (*C*H = CH_2_), 147.3 (N*C*H = C), 166.2 (C = O). EIMS (m/z, %) 243 (M^+^, 2), 197 (C_9_H_11_NO_4_^+^, 100). HRMS: calcd for C_11_H_17_NO_5_: 243.11067; found: 243.10985.

### General procedure for the thermal rearrangements of isoxazolinones

In a round bottom flask, the isoxazolinones (**8a-e**) dissolved in *o*-dichlorobenzene (ca. 0.04 – 0.05 M) are heated until total consumption of the starting material (TLC control). After solvent evaporation the products are purified by flash chromatography.

#### *Rearrangement of the 3,4-diphenyl-2-(prop-2′-yn-1′-yl)isoxazolin-5-one (8a)*

Following the general procedure, 3,4-diphenyl-4-(prop-1′,2′-dien-1-yl)isoxazolin-5-one (**9a**) was obtained by heating **8a** (10 mg) at 60°C for 30 m, followed by CC (silica; Et_2_O / *n*-hexane 1:1), as an oil (75%): IR (film, cm^-1^): 1965 (C = C = C), 1798 (C = O). ^1^H NMR (400 MHz CDCl_3_) *δ* 4.88 (1H, dd, *J* = 6.6, 12.1 Hz, CH = C = C*H*_2_), 5.04 (1H, dd, *J* = 6.6, 12.1 Hz, CH = C = C*H*_2_), 5.80 (1H, t, *J* = 6.6 Hz, *H*C = C = CH_2_), 7.30 – 7.58 (10H, m, Ar–H). ^13^C NMR ( 100.62 MHz CDCl_3_) *δ* 57.8 [N = C(Ph)*C*], 80.6 (CH = C = *C*H_2_), 88.0 (*C*H = C = CH_2_), 126.9 (Ar), 127.1 (Ar), 127.7 (Ar), 128.8 (Ar), 129.2 (Ar), 129.6 (Ar), 131.6 (Ar), 134.3 (Ar), 166.6 (N = C), 175.1 (C = O), 203.0 [CH = *C* = C(CH_3_)_2_]. EIMS (m/z, %): 275 (M^+^, 19), 231 ([M-CO_2_]^+^, 100), 172 (89), 128 (99), 102 (89), 77 (82). HRMS: calcd for C_18_H_13_NO_2_: 275.09463; found: 275.09556.

#### *Rearrangement of 3,4-diphenyl-2-(2′-methylbut-3′-yn-2′-yl)isoxazolin-5-one (8b)*

Following the general procedure, 3,4-diphenyl-4-(3′-methylbut-1′,2′-dien-1′-yl)isoxazolin-5-one (**9b**) was obtained by heating **8b** at 60°C for 10 m, followed by CC (silica; Et_2_O / *n*-hexane 1:1), as a colourless solid (85%): mp 77–80 (Et_2_O / *n*-hexane). IR (KBr, cm^-1^): 1969 (C = C = C), 1790 (C = O). ^1^H NMR (400 MHz CDCl_3_) *δ* 1.37 (3H, s, CH_3_), 1.62 (3H, s, CH_3_), 5.65 (1H, t, *J* = 2.6 Hz, HC = C = C), 7.28 – 7.54 (10H, m, Ar–H). ^13^C NMR (100.62 MHz CDCl_3_) *δ* 19.3 (CH_3_), 19.7 (CH_3_), 58.5 [N = C(Ph)*C*], 86.6 [*C*H = C = C(CH_3_)_2_], 102.2 (CH = C = *C*(CH_3_)_2_), 127.0 (Ar), 127.7 (Ar), 128.1 (Ar), 128.5 (Ar), 128.9 (Ar), 129.4 (Ar), 129.7 (Ar), 131.2 (Ar), 132.2 (Ar), 134.7 (Ar), 167.2 (N = C), 177.5 (C = O), 203.5 (CH = *C* = C(CH_3_)_2_). EIMS (m/z, %) 303 (M^+^, 1), 288 ([M-CH_3_]^+^, 29), 259 ([M-CO_2_]^+^, 53), 244 (C_18_H_14_N^+^, 50), 200 (C_13_H_12_O_2_^+^, 100), 141 (C_11_H_9_^+^, 71), 89 (C_7_H_5_^+^, 20), 77 (C_6_H_5_^+^, 76).

#### *Rearrangement of 2-allyl-3-methyl-4-phenylisoxazolin-5-one (8c)*

Following the general procedure, 4-allyl-3-methyl-4-phenylisoxazolin-5-one (**9c**) was obtained by heating **8c** at 180°C for 7 h, followed by CC (silica; Et_2_O / *n*-hexane 1:1), as a viscous oil (95%): IR (film, cm^-1^): 1791 (C = O), 1643 (C = N). ^1^H NMR (400 MHz CDCl_3_) *δ* 1.95 (3H, s, CH_3_), 2.85 (1H, dd, *J* = 7.8, 13.7 Hz, C–CH_2_), 3.10 (1H, dd, *J* 6.7, 13.7 Hz, C–CH_2_), 5.25 (1H, d, *J* = 10.1 Hz, C*H*_2_ = CH), 5.30 (1H, d, *J* = 16.9 Hz, C*H*_2_ = CH), 5.60 (1H, m, CH_2_ = C*H*), 7.20 – 7.44 (5H, m, Ar–H). ^13^C NMR (100.62 MHz CDCl_3_) *δ* 12.4 (CH_3_), 36.4 (C–*C*H_2_), 59.3 (*C*–CH_2_), 121.4 (*C*H_2_ = CH), 126.2 (Ar), 127.3, 127.8, 128.9, 129.5, 129.7, 133.3 (CH_2_ = *C*H), 168.3 (C = N), 178.7 (C = O). EIMS (m/z, %): 215 (M^+^, 100), 116 (15), 115 (40), 77 (30). HRMS: calcd for C_13_H_13_NO_2_: 215.09463; found: 215.09414.

#### *Rearrangement of 2-allyl-3,4-diphenylisoxazolin-5-one (8d)*

Following the general procedure, 4-allyl-3-methyl-3,4-phenylisoxazolin-5-one (**9d)** was obtained by heating **8d** at 180°C for 5 h, followed by CC (silica; Et_2_O / *n*-hexane 1:1), as a pale yellow solid (97%): mp 71–72°C. IR (KBr, cm^-1^): 1800 (f, C = O), 1644 (C = N).^1^H NMR (400 MHz CDCl_3_) *δ* 3.03 (1H, dd, *J* = 7.2, 13.2 Hz, C–CH_2_), 3.32 (1H, dd, *J* = 7.6, 13.2 Hz, C–CH_2_), 5.25 (1H, d, *J* = 16,9 Hz, C*H*_2_ = CH), 5.14 (1H, d, *J* = 10.1 Hz, C*H*_2_ = CH), 5.54 (1H, m, CH_2_ = C*H*), 7.33 – 7.52 (10H, m, Ar–H). ^13^C NMR (100.62 MHz CDCl_3_) *δ* 37.5 (C–*C*H_2_), 58.8 (*C*–CH_2_), 121.8 (*C*H_2_ = CH), 126.3 (Ar), 127.2 (Ar), 127.4 (Ar), 128.9 (Ar), 129.0 (Ar), 129.4 (Ar), 129.7 (Ar), 131.7 (CH_2_ = *C*H), 134.4 (Ar), 167.1 (N = C), 169.9 (C = O). EIMS (m/z, %): 277 (M^+^, 100), 236 ([M-C_3_H_5_]^+^, 12), 232 (21), 178 (49), 117 (51), 103 (35), 77 (30). HRMS: calcd for C_18_H_15_NO_2_: 277.11028; found: 277.10956.

#### *Rearrangement of the ethyl 2-allyl-5-oxo-2,5-dihydroisoxazole-4-carboxylate (8e)*

Following the general procedure, ethyl 2-cyanopent-4-enoate (**12**) was obtained by heating **8e** at 180°C for 5 h, followed by CC (silica; Et_2_O / *n*-hexane 1:1), as a colourless oil [13] (80%): IR (film, cm^-1^): 2266 (-C≡N), 1750 (C = O). ^1^H NMR (400 MHz CDCl_3_) *δ* 1.32 (3H, t, *J* = 7.1 Hz, OCH_2_C*H*_3_), 2.69 (2H, m, CHC*H*_2_CH), 3.56 (1H, t, *J* = 6.8 Hz, NC–*C*H), 4.27 (2H, q, *J* = 7.1 Hz, OC*H*_2_CH_3_), 5.25 (2H, m, C*H*_2_ = CH), 5.83 (1H, m, CH_2_ = C*H*).

### Reaction of isoxazolinones with base

#### *Reaction of 2-allyl-3,4-diphenylisoxazolin-5-one (8d)*

To a stirred solution of compound **8d** (80 mg, 0.289 mmol) in dry 1,4-dioxane (2 mL) at RT under argon, potassium ethoxide (1.5 eq., 36.5 mg, 0.434 mmol) and 18-crown-6 ether (114.7 mg, 0.434 mmol) were added. After 10 m the reaction was found complete (TLC control: silica; Et_2_O / *n*-hexane 3:1), the mixture was neutralized with aq. 0.5 N AcOH and the solvent removed under reduced pressure. The crude was diluted with Et_2_O (5 mL) and washed with distilled water (2 mL). The organic layer was dried over anhydrous sodium sulphate, filtered and concentrated under reduced pressure. Purification by CC (silica; Et_2_O / *n*-hexane 3:1) afforded 2-(2′-ethoxyethyl)-4,5-diphenyl-2,3-dihydro-1,3-oxazin -6-one (**14**) (23.3 mg, 25%) as a yellow oil: IR (film, cm^-1^): 3257 (NH), 1694 (C = O), 1600, 1585, 1553. ^1^H NMR (400 MHz CDCl_3_) *δ* 1.05 (3H, t, J = 7.0 Hz, OCH_2_C*H*_3_), 2.22 (2H, m, CHC*H*_2_), 3.44 (2H, m, OC*H*_2_CH_3_), 3.62 (1H, m, OC*H*_2_CH_2_), 3.77 (1H, m, OC*H*_2_CH_2_), 5.47 (1H, q, *J* = 4.9 Hz, C*H*CH_2_), 6.46 (1H, d, *J* = 4.1 Hz, NH – exchange D_2_O), 7.02 – 7.24 (10H, m, Ar–H). ^13^C NMR (100.62 MHz CDCl_3_) *δ* 15.0 (OCH_2_*C*H_3_), 32.0 (OCH_2_*C*H_2_), 65.7 (O*C*H_2_CH_3_), 66.6 (O*C*H_2_CH_2_), 81.6 (N–CH–O), 103.9 (N–C = *C*–CO), 126.1 (Ar), 127.6 (Ar), 128.2 (Ar), 129.8 (Ar), 130.2 (Ar), 131.4 (Ar), 133.9 (Ar), 134.9 (Ar), 155.5 (N–C = *C*–CO), 166.3 (C = O). EIMS (m/z, %) 323 (M^+^, 100), 250 (C_16_H_12_NO_2_^+^, 90), 178 (C_14_H_10_^+^, 99), 77 (C_6_H_5_^+^, 43). HRMS: calcd for C_20_H_21_NO_3_: 323.15214; found: 323.15254; and deoxybenzoin (**15**, R = H) (25.5 mg, 45%) as a colourless solid: mp 55–56°C (lit.: [25] mp 55–56°C). IR (KBr, cm^-1^): 1686 (C = O). ^1^H NMR (400 MHz CDCl_3_) *δ* 4.29 (2H, s, CH_2_), 7.24 – 8.04 (10 H, m, Ar–H). After the reaction in a parallel run was completed, glacial CH_3_COOD (3.0 eq.) was added to the reaction followed by H_2_O (2 mL). The reaction mixture was diluted with Et_2_O (5 mL) and washed with an aqueous solution of 1M NaHCO_3_. The aqueous layer was extracted with Et_2_O (3 × 5 mL), the combined organic extracts were dried over anhydrous sodium sulphate, filtered and concentrated. Purification by CC (silica; Et_2_O / *n*-hexane 1:1) afforded mono-deuterated deoxybenzoin (**15**, R = D) (37.5 mg, 66%): ^1^H NMR (400 MHz CDCl_3_) *δ* 4.29 (1H, s, C*H*D), 7.23 – 8.06 (10 H, m, Ar–H).

#### *Reaction of ethyl 2-allyl-5-oxo-2,5-dihydroisoxazole-4-carboxylate (8e)*

To a stirred solution of compound **8e** (50 mg, 0.254 mmol) in dry 1,4-dioxane (1 mL) at RT under argon, was added potassium ethoxide (32 mg, 0.38 mmol, 1.5 eq.) and 18-crown-6 ether (100 mg, 0.38 mmol). On completion of the reaction (5 h, TLC control: silica; Et_2_O), the solvent was removed under reduced pressure and the crude purified by cc (silica; Et_2_O). *N*-Allyl-malonamic acid ethyl ester (**13)** was obtained as a white solid (34.7 mg, 80%): mp 40–41°C (Et_2_O). IR (KBr) ν_max_ (cm^-1^): 3299 (NH), 1740 (C = O ester), 1659 (C = O amide). ^1^H NMR (400 MHz CDCl_3_) *δ* 1.22 (3H, t, *J* = 7.1, OCH_2_C*H*_3_), 3.26 (2H, s, COC*H*_2_COOEt), 3.84 (2H, t, *J* = 5.6 Hz, NH-C*H*_2_), 4.13 (2H, q, *J* = 7.1 Hz, OC*H*_2_CH_3_), 5.07 (1H, dd, *J* = 1.1, 10.3 Hz, C*H*_2_ = CH), 5.14 (1H, dd, *J* = 1.3, 17.1 Hz, C*H*_2_ = CH), 5.80 (1H, m, CH_2_ = C*H*), 7.29 (1H, bs, NH exchange D_2_O). ^13^C NMR (100.62 MHz CDCl_3_) *δ* 14.0 (COOCH_2_*C*H_3_), 41.3 (CO*C*H_2_COOEt), 41.8 (NH*C*H_2_), 61.5 (COO*C*H_2_CH_3_), 116.2 (*C*H_2_ = CH), 133.7 (CH_2_ = *C*H), 165.0 (NH*C* = O), 169.3 (*C*OOEt). EIMS (m/z, %): 171 (M^+^, 10), 56 (C_3_H_6_N^+^, 100). HRMS: calcd for C_8_H_13_NO_3_: 171.08954; found: 171.08894.

#### *Reactions of 2-(2′-methylbut-3′-yn-2′-yl)-3,4-diphenylisoxazolin-5-one (8b)*

##### With 1.5 eq. of EtOK

To a stirred solution of compound (**8b**) (15 mg, 0.055 mmol) in dry 1,4-dioxane (0.25 mL), under argon, was added potassium ethoxide (1.5 eq., 7.0 mg, 0.083 mmol) and 18-crown-6 ether (21.8 mg, 0.083 mmol). On completion of the reaction (10 min) (TLC control: silica; Et_2_O / *n*-hexane 3:1), the mixture was neutralized with aq. AcOH 0.5 N and the solvent removed under reduced pressure. The crude was diluted with Et_2_O (5 mL) and washed with destilled H_2_O (2 mL). The organic layer was dried over anhydrous sodium sulphate, filtered and concentrated under reduced pressure. Purification by CC (silica; Et_2_O / *n*-hexane 3:1) afforded 4-(3′-methylbut-1′,2′-dienyl-1′-yl)-3,4-diphenylisoxazolin-5-one (**9b**) (4.5 mg, 30%). After 30 min of reaction, no rearrangement product could be still detected by TLC.

##### With 0.1 eq. of EtOK

Using the previous conditions with potassium ethoxide (0.1 eq.) and 18-crown-6 ether (0.1 eq.), the reaction after 5 days yielded **9b** (55%).

##### With LDA

To a stirred solution of **8b** (1 eq.) in dry 14-dioxane, under argon, were added LDA (1.5 eq., from a 1.47 M solution) and 12-crown-4 ether (1.5 eq.). The reaction mixture was stirred for 48 h. TLC control (silica; Et_2_O / *n*-hexane 3:1) showed no reaction and the starting material was recovered yield (5.4 mg, 90%).

##### With tert-BuLi

To a stirred solution of **8b** (1 eq.) in dry 1,4-dioxane, under argon, were added *tert*-BuLi (2 eq., from an hexane solution 0.8 M, 0.066 mmol) and 12-crown-4 ether (2 eq.). The reaction mixture was stirred for 70 h. TLC control (silica; Et_2_O / *n*-hexane 3:1) showed no reaction and the starting material was recovered (9.5 mg, 95%).

## Endnote

^a^ Web Table (which contains energy profiles, molecular coordinates of intermediates and transition states as well as normal mode animations) is available via the HTML version of the article.

## Competing interests

The authors declare that they have no competing interest.

## Supplementary Material

Additional file 1: Table S1 Calculated relative energies for intermediates and transition states relating to Scheme 6. Click here for file
