# Supplementary material for: N-heteroatom substitution effect in 3-aza-cope rearrangements
Source: Chem Cent J. 2013 May 28;7:94. doi: 10.1186/1752-153X-7-94 (PMC3702470; doi:10.1186/1752-153X-7-94)
Supplement: Additional file 1: Table S1 — Calculated relative energies for intermediates and transition states relating to Scheme 6. [file 1752-153X-7-94-S1.doc]

# Additional files

### Web Table 1 - Calculated relative energies for intermediates and transition states relating to Scheme 6.

| # | System | Relative free energy {ΔG^‡^}^a^ (kcal.mol^-1^) | Digital Repository^b^ |
| --- | --- | --- | --- |
| 1 | **8e + ethoxide** | 8.0 | [10042/to-11762](http://hdl.handle.net/10042/to-11762) |
| 2 | **8e-1** | 0.0 | [10042/to-11748](http://hdl.handle.net/10042/to-11748) |
| 3 | **8e-2** | 17.0 | [10042/to-11747](http://hdl.handle.net/10042/to-11747) |
| 4 | **8e-1 TS1** | 57.2 | [10042/to-8533](http://hdl.handle.net/10042/to-8533) |
| 5 | **8e** **TS2 first step** | 13.5 | [10042/to-8577](http://hdl.handle.net/10042/to-8577) |
| 6 | **8e** **TS2 intermediate** | 8.3 | [10042/to-8576](http://hdl.handle.net/10042/to-8576) |
| 7 | **8e** **TS2 second step** | 5.9 | [10042/to-8574](http://hdl.handle.net/10042/to-8574) |
| 8 | **8e-4** | -35.2 | [10042/to-11749](http://hdl.handle.net/10042/to-11749) |
| 9 | **8e-4** **TS** | 7.2 | [10042/to-11763](http://hdl.handle.net/10042/to-11763) |
| 10 | **8e-5** | -20.1 | [10042/to-11752](http://hdl.handle.net/10042/to-11752) |
| 11 | **8e-6** | -33.7 | [10042/to-11753](http://hdl.handle.net/10042/to-11753) |
| 12 | **8e-8** | -56.8 | [10042/to-11754](http://hdl.handle.net/10042/to-11754) |

^a^Geometries optimized at the ωB97XD/6-311G(d,p)/SCRF(CPCM, Solvent=ethanol) level. ^b^The Web Table version requires a Java-enabled browser to view 3D models; full details of calculations available *via* the link to the digital repository entry indicated. The interactive version of this table is available via the link www.??? (journal staff to add link).
